# Epitome of Surgery

**Published:** 1889-03

**Authors:** 


*Epitome of Surgery*. By RIDLEY DALE, M.D. Pp. 498. London: H. K. LEWIS. 1889.

It is rare indeed that synopses, or epitomes, or students' guides are worthy of commendation. In such works elision is usually made the substitute for condensation, and system and classification, the prime features in all works intended as guides for the student, are either ignored or imperfectly carried out.

It has given us real pleasure to find an exception to these general rules. The work before us provides a really good synopsis of the leading principles in surgery; well-arranged, judiciously balanced, and tersely but clearly described. Printed in small type, two columns in the page, there is a large amount of information in the 500 octavo pages which go to form the book. The student should derive much advantage from the careful perusal of this work before presenting himself for examination.

